# Microbial Contamination of Orthodontic Buccal Tubes from Manufacturers

**DOI:** 10.3390/ijms11093349

**Published:** 2010-09-16

**Authors:** Kathiravan Purmal, Shenyang Chin, John Pinto, Wai-Fong Yin, Kok-Gan Chan

**Affiliations:** 1 Department of General Dental Practice and Oral and Maxillofacial Imaging, Faculty of Dentistry, University of Malaya, 50603 Kuala Lumpur, Malaysia; E-Mail: drkathi@um.edu.my; 2 Division of Genetics and Molecular Biology, Institute of Biological Sciences, Faculty of Science, University of Malaya, 50603 Kuala Lumpur, Malaysia; E-Mails: shenyang86@yahoo.com (S.C.); jmp_forever@yahoo.com (J.P.); yinwaifong@yahoo.com (W.-F.Y.)

**Keywords:** Acinetobacter calcoaceticus, Micrococcus luteus, oral cavity, orthodontic buccal tubes, Staphylococcus haemolyticus, sterilization

## Abstract

This study aimed to test the sterility of new unused orthodontic buccal tubes received from manufacturers. Four different types of buccal tubes were used straight from the manufactures package without any additional sterilizing step. Of these buccal tubes tested, three genera of bacteria, implicated as opportunistic pathogens, namely *Micrococcus luteus*, *Staphylococcus haemolyticus* and *Acinetobacter calcoaceticus* were recovered from these buccal tubes. Our data showing microbial contamination on buccal tubes highlights the need of sterilization before clinical use. We also suggest that manufacturers should list the sterility state of orthodontic buccal tubes on their packaging or instructions stating the need for sterilization.

## 1. Introduction

Human oral flora is a unique habitat which consists of at least 400 to 700 different bacterial species [1]. Oral bacteria represent a complex and dynamic community and are responsible for, *inter alia*, two human oral infectious diseases: caries and periodontal disease [2]. Orthodontic buccal tubes are used in orthodontic treatment to facilitate the movement of teeth along the archwire. They are attached to the molars but come in contact with intact mucous membranes. Sometimes they lacerate the mucosa and cause injury. The design of the buccal tubes and their various welded attachments present potential areas for bacterial adherence. Therefore, these buccal tubes can be considered as semi-critical medical instruments that require a high level of disinfection [3]. Lucas *et al.* [4] have demonstrated that orthodontic treatment procedures can cause bacteraemia by aerobic and anaerobic bacteria.

There is a clear guideline on the sterilization requirements for any instrument that comes into contact with human tissues, including those of dental use [5]. Sterilization can be achieved primarily by high heat treatment such as autoclave or irradiation, with the aim of destroying all forms of microorganisms to reduce the introduction of or spread of infectious diseases. This is a crucial step, including the sterilization of dental instrument such as orthodontic buccal tubes.

Although the dental literature presents an abundance of studies on microbial adherence onto orthodontic appliance and utilities [6–9], none of the studies have looked into buccal tubes that are new from the manufacturer. The manufacturers do not state on the packaging that the buccal tubes should be sterilized before clinical application. A review of the literature found no investigation to determine the presence of microbial contamination among a wide variety of new buccal tubes received from the manufacturer. Therefore, this study was designed to ascertain the sterility of new buccal tubes and to identify the presence of any pathological microorganisms, as some organisms are known to survive for more than a month in dry conditions and resist heat up to 60 °C [6].

## 2. Results and Discussion

**Figure 1:**
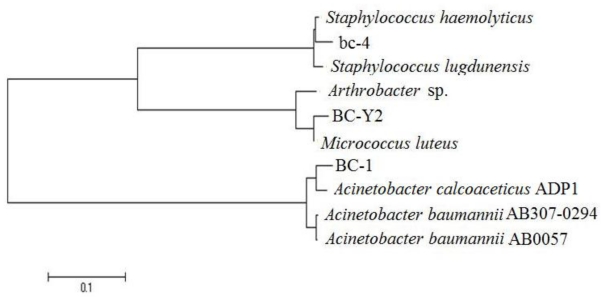
Phylogenetic relationship of the bacteria isolated from the buccal tubes. Small-subunit rRNA-based evolutionary tree showing the phylogenetic position of strains BC-Y2, bc-4 and BC-1. A total of 538 unambiguously aligned nucleotides were used for analysis using Bootstrap value of 1,000. The bar represents evolutionary distance as 0.1 changes per nucleotide position. GenBank accession numbers in parentheses: *Acinetobacter baumannii* AB0057 (gi213155370:56013-57570), *Acinetobacter calcoaceticus* ADP1 (gi50083297:18416-19945), *Acinetobacter baumannii* AB307-0294 (gi215481761:18347-19871), *Staphylococcus lugdunensis* HKU09-01 (gi289549371:916199-917737), *Staphylococcus haemolyticus* JCSC1435 (gi70725001:879834-881387), *Micrococcus luteus* NCTC 2665 (gi239916571: 419927-421558), *Arthrobacter* sp. FB24 (gi116668568:792344-793860). For strains BC-Y2, bc-4 and BC-1, see text.

Three strains (labeled as BC-Y2, bc-4 and BC-1) were isolated from the buccal tubes. Six buccal tubes were found to be contaminated by bacteria. No bacteria were isolated from saline without the buccal tubes (data not shown). Based on 16S rRNA genes phylogenetic analyses, the identities of these bacterial strains (BC-Y2, bc-4 and BC-1) are: *Micrococcus luteus*, *Staphylococcus haemolyticus* and *Acinetobacter calcoaceticus*, respectively (Figure 1). The16S rRNA genes sequences for BC-Y2, bc-4 and BC-1 have been deposited in GenBank with the following accession numbers: GU370962, GU300765 and GU370964, respectively.

The evolutionary history was inferred using the Neighbor-Joining method [10]. The bootstrap consensus tree inferred from 1000 replicates [11] is taken to represent the evolutionary history of the taxa analyzed [11]. Branches corresponding to partitions reproduced in less than 50% of the bootstrap replicates are collapsed. The tree is drawn to scale, with branch lengths in the same units as those of the evolutionary distances used to infer the phylogenetic tree. The evolutionary distances were computed using the Kimura 2-parameter method [12] and are in the units of the number of base substitutions per site. All positions containing gaps and missing data were eliminated from the dataset (complete deletion option). There were a total of 538 positions in the final dataset. Phylogenetic analyses were conducted in MEGA4 [13].

Our study determined that buccal tubes received from manufacturers were biologically contaminated. However, the present work is not intended as a comprehensive survey for three reasons, namely: (1) Only four sets of buccal tubes from three manufacturers were tested; (2) only LB-agar was used for isolation of bacteria whereby most uncultivable bacteria will be missed; (3) the present study employed only one set of culture conditions where no attempt were made to isolate fungi, viruses and anaerobic bacteria in the present study. It must be pointed out that the present work is not aimed to determine the spectrum of microorganisms (such as viruses and fungi) and the microbial load on the buccal tubes. Neither do we attempt to identify from which manufacturer the contaminants are from because that was not our purpose. But rather, this work aimed specifically to study aerobic bacterial contamination by isolation and characterization of aerobic bacteria present on the surface of the available buccal tubes. This report established that some buccal tubes received from the manufacturers were contaminated with viable microorganisms. Contaminated buccal tubes may serve as a source for the subsequent cross-contamination of dental appliances; it will also result in the direct inoculation of micro-organisms into the mouths of patients.

Three manufacturers of the buccal tube types used in our study did not have any labeling with respect to sterility of the product. The packaging labels also did not have any suggestion whether these buccal tubes should be sterilized before clinical use. All these buccal tubes were packaged in a sealed plastic container; however, the majority of buccal tubes were not protected from the external environment by their packaging. Therefore, it is possible that contamination occurred during the manufacturing process, packaging and during transport from the manufacturers to the dental clinics or from repackaging by the local distributors. Metal orthodontic brackets bonded to the premolars were analyzed by scanning electron microscopy, indicating the presence of bacteria, predominantly streptococci, around the base of brackets [14], but no bacteria characterization was performed in that report. Another study reported that *Streptococcus mutans* were present in higher levels in patients with conventional and self-ligating brackets [15].

In the present work, the first microorganism isolated, *M. luteus*, is an obligate aerobe, that has been reported as the most common commensal species of *Micrococcus* found on human skin [16]. A previous study also reported that *M. luteus* can enter a profound dormancy period for 34,000 to 170,000 years in an oligotrophic environment such as amber [17]. *M. luteus* have numerous adaptations for survival in extreme, nutrient-poor environments, and *M. luteus* can be resuscitated from a dormancy state by a secreted protein called resuscitation-promoting factor [18]. Although human skin is now considered to be a primary habitat of the bacterium, and it has also been detected in water and soil, the mucous membranes, including the buccal cavity [19], septic arthritis [20], septic shock [21], meningitis [22] and pneumonia [23]. Considering all the reported work on *M. luteus*, this leads us to speculate that the reasons for the presence of *M. luteus* on the buccal tube are due to human contamination and its profound ability to survive even in nutrient-depleted condition.

The second microorganism found was *Acinetobacter calcoaceticus*. Although mostly found in the soil, *Acinetobacters* are part of the human skin flora also [24]. *A. calcoaceticus* is reported to be an opportunistic pathogen causing serious nosocomial diseases [25]. It has also been reported that *Acinetobacter* sp. has been recovered from used dental pumice [26].

The third microorganism identified, *S. haemolyticus*, is among the normal skin flora, and commonly isolated from the axillae, perineum and inguinal areas of humans [27]. Since this bacterium can be found among the normal skin flora, this suggests its origin in the buccal tubes can originate from contacts with the human skin or fingers. However, *S. haemolyticus* represents the second most commonly isolated bacteria recognized as important nosocomial pathogens [28,29]. Recently, multiple drug resistance has been documented in *S. haemolyticus* [30].

As all of the bacterial species cultured from the buccal tubes in this study are commonly associated with human being, we postulate that the presence of these bacteria are due to unhygienic practices of staff at the production line during manufacturing and packaging. We also speculate that the non-sterile packaging and improper handling of packages can also be a source of the bacterial contamination especially during logistics and storage. Lastly, the time of exposure of the buccal tubes upon being received in the clinic may explain the presence of these bacteria because these bacteria are common causes of nosocomial infections. These bacteria could have been acquired via exposure to the clinic environment. Our work highlights the need to consider sterilization of these buccal tubes as some of the bacteria isolated, such as *A. calcoaceticus*, is well recognized as a nosocomial species. Its long survival on a dry surface may be an additional factor aiding its transmission in hospitals [31] and suggests that more attention be paid to environmental surfaces including the buccal tubes as a source of significant nosocomial pathogens.

To our knowledge, no buccal tube sources have been identified in an oral health-care facility from which all three of these bacteria have been isolated. The potential spread of these organisms implicated as opportunistic in the dental operatory may pose a potential risk, and should pose a serious concern to dentists. This may increase the incidence of nosocomial bacteria and may cause serious implications on oral health.

## 3. Materials and Methods

### 3.1. Buccal Tube Samples

Four different types of buccal tubes from three manufacturers were selected for testing (Table 1). Upon inspection, there was no packaging information regarding sterility from any of the manufacturers. The original packages of the buccal tubes were opened using sterile latex gloves and the buccal tubes picked up with sterile tweezers and transferred to a sterile container. Six buccal tubes from each of the four types were tested (*N* = 24).

### 3.2. Isolation of Microorganisms

Each buccal tube was submerged in sterile saline (10 mL) in a sterile plastic tube and vortexed vigorously for 5 min. The resulting saline (100 μL) was serially diluted (ten-fold) with Luria Bertani (LB) broth and spread onto LB agar and incubated at 37 °C for 24–48 h. Pure colony was obtained by repeated streaking on LB agar and incubated at 37 °C for 24 h.

### 3.3. DNA Extraction, PCR and Sequencing

Extraction of bacterial genomic DNA was performed according to previously reported work [11]. The DNA extracted was subjected to PCR using the primers 27f (5′-AGAGTTTGATCMTGGCTCAG-3′) and 1525r (5′-AAGGAGGTGATCCAGCC-3′) as forward and reverse primers, respectively [32], at an annealing temperature of 55 °C, to selectively amplify the 16S rRNA gene region that is approximately 1500 bp in length. Purification and sequencing of PCR products were carried out according to published criteria [11]. All sequences obtained have been deposited in GenBank.

### 3.4. Analysis of Bacterial 16S rRNA Sequences

Alignment, editing and phylogenetic analysis of nucleotides sequences were performed according to the reported method [33]. Construction of phylogenetic tree was done using MEGA 4 [13].

## 4. Conclusions

Some buccal tubes received from the manufacturers were contaminated with viable aerobic bacteria, potentially nosocomial species. It is strongly suggested that buccal tubes should be sterilized by autoclave before clinical usage.

## Figures and Tables

**Table 1:** Types of buccal tubes from various manufactures.

Group	Type of Buccal	Tube Manufacturer	Prescription
A	Lower right molar single tube	American Othodontics, Sheboygan, Wis	MBT
B	Lower right molar single tube—small base	3M Unitek, Monrovia Calif	MBT
C	Lower right molar single tube—large base	3M Unitek, Monrovia Calif	MBT
D	Lower right molar single tube	Hangzhou Dentop, China	MBT

MBT = McLaughlin, Bennett and Trevisi prescription.
